# JC Virus Leuko-Encephalopathy in Reduced Intensity Conditioning Cord Blood Transplant Recipient with a Review of the Literature

**DOI:** 10.4084/MJHID.2012.043

**Published:** 2012-06-20

**Authors:** Jean El-Cheikh, Sabine Fürst, Francois Casalonga, Roberto Crocchiolo, Luca Castagna, Angela Granata, Claire Oudin, Catherine Faucher, Pierre Berger, Anthony Sarran, Didier Blaise

**Affiliations:** 1 Unité de Transplantation et de Thérapie Cellulaire (U2T), Institut Paoli-Calmettes, Marseille, France; 2 Département d’Onco-Hématologie, Institut Paoli-Calmettes, Marseille, France; 3 Département de Radiologie, Institut Paoli-Calmettes, Marseille, France; 4 Département de Microbiologie, Institut Paoli-Calmettes, Marseille, France

## Abstract

We report here the case of progressive multifocal leukoencephalopathy (PML) related to human polyomavirus JC (JCV) infection after an allogeneic transplantation with umbilical cord blood cells in 59-year-old woman with follicular Non Hodgkin lymphoma. She presented with dysphagia and weakness; magnetic resonance imaging demonstrated marked signal abnormality in the sub-cortical white matter of the left frontal lobe and in the posterior limb of the right internal capsule. Polymerase chain reaction (PCR) analysis of the cerebrospinal fluid (CSF) was positive for John Cunningham (JC) virus. JC viral DNA in the CSF was positive, establishing the diagnosis of PML. Brain biopsy was not done. Extensive investigations for other viral infections seen in immuno-compromised patients were negative. The patient’s neurologic deficits rapidly increased throughout her hospital stay, and she died one month after the diagnosis. These findings could have practical implications and demonstrate that in patients presenting neurological symptoms and radiological signs after UCBT, the JCV encephalitis must be early suspected.

## Introduction

Progressive multifocal leukoencephalopathy (PML) is a rapidly progressive demyelinising disorder of the central nervous system almost exclusively encountered in immuno-compromised individuals.^1^–^4^ It is caused by reactivation of the John Cunningham virus (JCV) under conditions of cellular immuno-compromise such as those encountered in patients with acquired immunodeficiency syndrome (AIDS), patients with hematologic and solid organ malignancies receiving chemotherapy, and transplant recipients under immuno-suppression.^1^,^2^ The interest in this disease has recently increased because of its association with natalizumab, a monoclonal antibody directed against α_4_ integrins that is used to treat Crohn’s disease^3^,^4^ and multiple sclerosis.^5^–^7^ Presently, there is no available structured series of JCV encephalitis in literature regarding allogeneic umbilical cord blood transplantation (UCBT), but just anecdotal cases have been reported. Although, there is no universally effective antiviral therapy against JCV and outcome is fatal in the majority of cases.

We hereby describe a rare case of PML, developing after UCBT and provide a comprehensive review of the literature in order to better define the epidemiological, clinical and therapeutic findings of this rare complication.

## Case report

Here, we describe a rare occurrence of PML, with a rapidly fatal outcome, in 59-year-old woman who underwent UCBT for Follicular Non Hodgkin Lymphoma in January 2011; she was in complete remission (CR) confirmed by the PET scan after 5 lines of treatment. At transplantation, no organ dysfunction was present.

The reduced intensity conditioning (RIC) regimen consisted of total body irradiation (2 Gys in 1 fraction), Fludarabine at 40 mg/m^2^/day for 5 days, and Cyclophosphamide at 50mg/Kg/day. A total nucleated cell dose of 3.3 10^7^/kg body weight was infused on day 0. The cord blood unit and the patient were HLA matched 4/6 with mismatch of locus A and B. Graft-versus-host disease (GVHD) prophylaxis consisted of cyclosporine A at 3 mg/kg/day, and Mycophenolate Mofetyl (MMF) at 500mgx 4/day, beginning on day -3. Neutrophil engraftment (>0.5 × 10^9^/L) occurred on day 26.

On day 35, the patient presented a skin grade 2 acute GVHD reaction, without other organ dysfunction, and 2 mg/kg methyl-prednisolone was started; a rapid clinically response was observed. On day 51, the patient developed CMV reactivation detected by quantitative polymerase chain reaction (PCR) in the peripheral blood with 1359 copies; and an antiviral treatment with Gancyclovir (Cymevan) 2.5 mg/kg/day was administrated after adaptation with her renal function.

On day 68, at the time of her outpatient visit, she developed confusion, short-term memory dysfunction, and altered mental status, with focal signs and abnormal Babinsky reflexes on the left, and lack of the force on the thighs lower limbs. A brain computed tomography (CT) scan demonstrated no specific findings. The patient was described as cachectic, alert, awake, and oriented to person, time, and place. Her speech was fluent; comprehension, naming, and repetition were intact. Examination of the cranial nerves showed mild drooping of the left angle of the mouth; all other cranial nerves were intact. Motor strength was 4+/5 on the left symmetrically in the upper and lower extremities and 5-/5 in the right extremities. Deep tendon reflexes were hyperactive, but no extensor plantar responses were recorded. The complete blood count and chemistry levels were normal, except for positive schizocytes. Blood urea nitrogen and serum creatinine levels were 15.9 mmol/L and 179 μmol/L, respectively. The diagnosis of thrombotic microangiopathy was made in the presence of haemolytic anemia, thrombocytopenia, renal failure and presence of schizocytes in biological assessment. The patient received corticotherapy at 1mg/kg/day, and 5 plasma exchanges were made. The cyclosporine was discontinued and MMF treatment was continued. We assisted rapidly to a response of her haemolytic anemia, improved thrombocytopenia but without improvement of her neurological troubles. With worsening mental status on day 83, a magnetic resonance imaging (MRI) of the brain after gadolinium injection showed a non-enhancing hypo-intensity Image in (T1- sequence) beyond the white matter.

However, after gadolinium injection showed an image in the axial (FLAIR sequence) showing hyper-intensity lesions in the white matter of the frontal lobes. There is no signal abnormality of the cortex. Note that there is no mass effect on the ventricular cavities or midline structures.

Furthermore the MRI in B1000 diffusion sequence showed a restriction of diffusion in the damage areas of the white matter.

On day 84, the patient’s mental status was stable, and a lumbar cerebrospinal fluid (CSF) examination revealed a white blood cell (WBC) count of 1 cells/L, a normal Glucose level of 3.89 mmol/L and a normal protein level of 389mg/L. the mycological (aspergillosis, Cryptococcus), and Gram staining and culture were negative. A specimen was sent for polymerase chain reaction (PCR) analysis for JC virus was positive in the CSF and the serum (negative for CMV, VZV, HHV-6, EBV and herpes simplex virus by PCR). A treatment with 5 mg/kg/week of Vistid (Cidofovir) was initiated on day 86 associated with Mefloquine (Lariam^®^) and Mirtazapine (Norset^®^). It was around this time that the patient’s general condition began to worsen. Acute deterioration of cognitive function occurred with confusion and autism. Because of CR of her GVHD; steroids were tapered and ultimately discontinued on day 97. An electroencephalogram demonstrated mild bi-frontal slowing, with diffuse brain damage, more prominent on the left than the right.

Re-examination of CSF on day 93 revealed a WBC count of 3 cells/mL, a protein level of 323 mg/L, Glucose level of 5.08 mmol/L. JCV was positive in CSF by PCR. HHV- 6, CMV, HSV, VZV, EBV, enterovirus were negatives by qualitative PCR assay. PCR of the CSF for JCV conducted in the same laboratory as previously was positive, establishing the diagnosis.

Throughout her hospital stay, the patient’s neurologic condition continued to deteriorate. Her course was marked by an episode of altered mental status, progressive dementia, motor weakness, declining visual acuity and worsening performance status. In the light of clinical deterioration no further exams was performed in respect to the family’s wish for no further intervention. She was continued to a skilled nursing facility in our unit, where she died one month after the diagnosis of PML, on day 110 after UCBT.

Unfortunately we did not perform a neuropathologic examination of the brain by autopsy in respect to the family’s wish.

## Discussion

PML was originally described in 1958 in two patients with chronic lymphocytic leukemia and one with Hodgkin lymphoma.^8^ The causative agent, the JC virus, was isolated in 1971 from the brain of a patient with Hodgkin’s disease, and the virus was named after him.^9^ With the advent of the HIV epidemic, PML was recognized as a major opportunistic infection of AIDS^10^,^11^ but with effective antiretroviral therapy, its incidence and attributable mortality rates have decreased.^12^,^13^

Most recently, interest in PML has been described by its association with natalizumab (Tysabri^®^), a promising new drug for the treatment of multiple sclerosis and Crohn’s disease.^1^,^12^,^14^ Other groups in whom PML has been described are patients with chronic lymphoid leukemia treated in particular with fludarabine.^15^ The occurrence of PML in non immuno-compromised patients is exceedingly rare. The clinical picture of the disease is known, as well as the microbiological investigations to be performed on CSF. On the contrary we have few experiences about therapy.

However, to our knowledge, the number of reports of JCV encephalitis on this issue is not numerous and there exist only a few histopathologic descriptions. Although older age has been associated with idiopathic CD4+ lymphocytopenia,^16^ and the latter has been associated with PML,^17^ our patient did not have a control for CD4+ lymphocytopenia. With her lymphocyte count ranging between 200 and 1300 lymphocytes per microliter, however, it is possible that she had transient CD4+ lymphocytopenia. The association of aging with CD4+ lymphocytopenia^18^,^19^ and the high JCV sero prevalence in adults^20^ should increase awareness about the possible diagnosis of PML in the appropriate clinical and radiographic setting.

JC virus disease is a rare infectious complication after allo-SCT.^21^ Studies have shown that HHV-6 infection occurs frequently in adult patients after UCBT,^22^ and that UCBT is a risk factor for HHV-6 encephalitis.^22^ There are no data in the literature on the frequency of JCV after UCBT.

Several reports have indicated that the CNS CMV infection in stem cell transplant patients was associated with HHV-6 virus encephalitis, in that there is evidence of high-level viremia, retinitis, or extra-neural involvement.^22^–^23^

Negative PCR results for JCV-DNA in the CSF have been described in patients with AIDS,^20^,^22^ and a correlation with active antiretroviral treatment has been hypothesized.^22^ The fact that a negative PCR result for JCV-DNA cannot completely exclude PML has raised the need for a new consensus terminology, in which immuno-suppressed patients with clinical and radiographic features consistent with PML and no other etiology should be considered as “possible PML”.^22^

In contrast, our patient and 2 others described in the literature^24^ had relatively low levels of virus loads in the peripheral blood and no clinical evidence of invasive JC virus disease outside the brain. While strategies to reverse immunodeficiency, such as discontinuation of immunosuppressive therapy, institution of antiretroviral therapy in HIV-positive patients, 22 plasma exchange and immuno-adsorption in natalizumab treated patients,^26^,^27^ work well in certain groups. Cytarabine, 22,28,29 Cidofovir,^22^,^30^,^31^ Topotecan^32^ and Mirtazapine^33^ have been investigated as therapeutic agents, mostly in patients with AIDS, with variable results and toxicities. A study to investigate the effects of Mefloquine in PML is currently ongoing.^34^

Recently, Fianchi L. et al. described an atypical presentation of PML in a multiple myeloma patient after auto-SCT successfully treated with i.v. immunoglobulin in addition to the combination therapy.^35^ A synergistic effect in this case could be hypothesized; with the immunomodulation and perhaps re-myelination by i.v. Immunoglobulin, block of viral cell entry by 5-HT2a receptor antagonist and inhibition of viral replication in cells by Mefloquine. This seemed to be an attractive option; however, the value of this approach remains to be determined in clinical trials.

In addition to the significant immune dysfunction associated with UCBT and high-dose corticosteroid treatment poor drug penetration to the CNS and low dosage of Gancyclovir and Cidofovir likely contributed to treatment failure in this case. Although no resistance data are available on this virus. Patients with delayed immune reconstitution and persistently low CD4 count may be at high risk for JCV encephalitis, in particular following augmentation of immuno-suppression for treatment of GVHD or in patients treated with rituximab (anti CD20).^36^,^37^ The early occurrence of encephalitis after UCBT may have an impact in association with microbiological and MRI findings. Interestingly, our patient developed JCV encephalitis following an episode of CMV reactivation and thrombotic microangiopathy syndrome.^38^,^39^ Several investigators have reported that JCV is associated with increased risk for invasive and symptomatic CMV disease.^40^,^41^ Although the most important risk factor of JCV encephalitis in our patient was her severely immuno-compromised status it is possible that the antecedent episode of CMV infection, the previous acute GVHD and the thrombotic microangiopathy, contributed to the fatal outcome of this patient. Early diagnosis of PML is, however, equally crucial to start adequate therapy before irreversible neurological damage has occurred.

In conclusion, these findings of JC virus encephalitis following RIC UCBT could have practical implications in patients presenting neurological symptoms and radiological signs after UCBT, the JCV encephalitis must be early suspected. Unfortunately inadequate drug penetration and the multi antiviral resistance as well as the severely immuno-suppressed state of this patient may have contributed to treatment failure.

## Funding

We would like to thank the Association pour la recherche sur le Cancer (ARC) (Pole ARECA) for their generous support of our research. Our group is supported by several grants from the French Ministry of Health as part of the Programme Hospitalier de Recherche Clinique (PHRC).

## Figures and Tables

**Figure 1:**
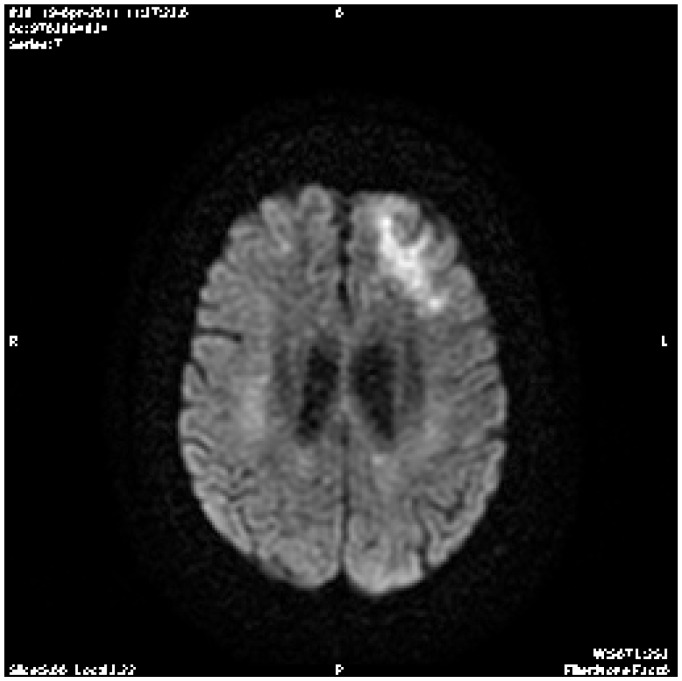
Brain magnetic resonance imaging (MRI) after gadolinium injection showed a non enhancing hypo-intensity Image in (T1- sequence) beyond the white matter.

**Figure 2:**
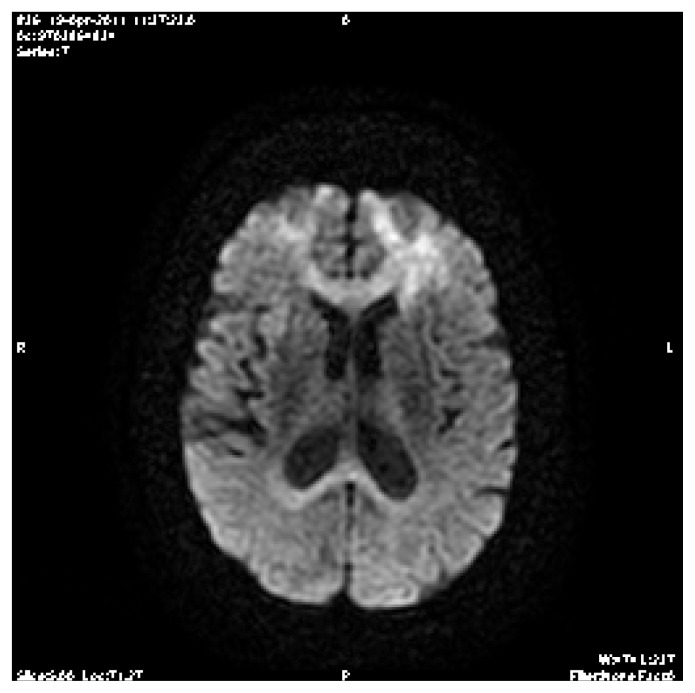
Brain magnetic resonance imaging (MRI) after gadolinium injection showed an image in the axial (FLAIR sequence) showing hyper-intensity lesions in the white matter of the frontal lobes. There is no signal abnormality of the cortex. Note that there is no mass effect on the ventricular cavities or midline structures.

**Figure 3:**
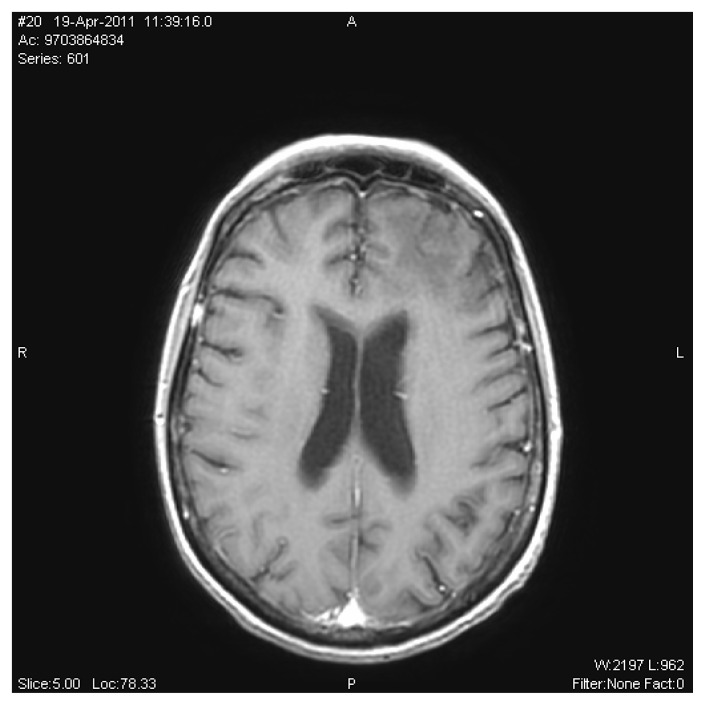
Brain magnetic resonance imaging (MRI) in B1000 diffusion sequence showed a restriction of diffusion in the damage areas of the white matter.
